# Supplementary material for: Evaluation of Genetic Testing in a Cohort of Diverse Pediatric Patients in the United States with Congenital Cataracts
Source: Genes (Basel). 2023 Feb 28;14(3):608. doi: 10.3390/genes14030608 (PMC10048176; doi:10.3390/genes14030608)
Supplement: Supplementary file 1 [file genes-14-00608-s001.zip › genes-2190704-supplementary.pdf]

Supplementary Table S1 (A-F) Genes tested for in 7 different commercially available targeted panels.

Table S1A. Prevention Genetics

FREE CATARACT PANEL	COMPREHENSIVE CATARACT PANEL
ABHD12, ADAMTSL4, ALDH18A1, BCOR, BEST1, BFSP1, BFSP2, CHMP4B, COL11A1, COL18A1, COL2A1, COL4A1, CRYAA, CRYAB, CRYBA1, CRYBA2, CRYBA4, CRYBB1, CRYBB2, CRYGC, CRYGD, CRYGS, CYP27A1, DYNC1H1, EPHA2, ERCC2, FAM126A, FOXE3, FTL, FYCO1, FZD4, GALE, GALK1, GALT, GCNT2, GJA3, GJA8, GLA, HSF4, LEMD2, LIM2, LONP1, MAF, MIP, MIR184, MYH9, NACC1, NDP, NF2, NHS, OPA3, P3H2, PAX6, PITX3, RAB3GAP1, RDH11, RECQL4, SC5D, SIL1, SLC16A12, TFAP2A, TGM3, UNC45B, VIM, WRN, XYLT2	ABCB6, ADAMTS10, AGK, AGPS, AKAP14, ALDH18A1, ALPL, B3GLCT, BCOR, BEST1, BFSP1, BFSP2, BMP4, BMP7, CAPN15, CCNP, CHMP4B, COL11A1, COL18A1, COL2A1, COL4A1, COL4A2, CRYAA, CRYAB, CRYBA1, CRYBA4, CRYBB1, CRYBB2, CRYBB3, CRYGB, CRYGC, CRYGD, CRYGS, CTDP1, CYP27A1, DHCR7, ELP4, EPG5, EPHA2, ERCC1, ERCC2, ERCC5, ERCC6, ERCC8, ESCO2, EVA1A, EYA1, FAM126A, FAM131A, FBN1, FOXC1, FOXE3, FTL, FYCO1, FZD4, GALK1, GCNT2, GJA1, GJA3, GJA8, GJC3, GLA, GNPAT, GPR160, GRWD1, HCCS, HMX1, HSF4, INPP5B, JAM3, KLHL21, LAMB1, LARGE1, LCT, LENG8, LIM2, LMX1B, LRP5, LTBP2, LTBP3, MAB21L1, MAF, MAN1C1, MAN2B1, MAP6D1, MECR, MEIS1, MIP, MIR184, MMP1, MSRA, MTMR7, MXRA8, MYH9, NDUFA1, NECTIN2, NECTIN3, NF2, NHS, NIPAL3, NRCAM, OCRL, OPA3, OTX2, P3H2, PAX6, PEAK1, PEX1, PEX10, PEX11B, PEX12, PEX13, PEX16, PEX2, PEX26, PEX3, PEX6, PEX7, PITX2, PITX3, PLD1, PLD3, POMT1, POMT2, PON2, PRKCI, PROX1, PRX, PTCH1, PTPRU, PXDN, RAB18, RAB3GAP1, RAB3GAP2, RECQL4, RHOBTB2, SC5D, SEC23A, SEMA3A, SIL1, SIPA1L3, SIX3, SIX5, SIX6, SLC16A12, SLC1A5, SLC25A13, SLC25A33, SLC25A40, SLC2A1, SLC33A1, SOX1, SOX2, SPINT2, SRD5A3, SREBF2, STEAP1, STEAP2, TACR1, TBC1D20, TDRD7, TMED3, TMEM114, TNPO1, TRAPPC6A, TTC14, UPF3B, VIM, VSX2, WFS1, YBX1

Table S1B. Invitae Panel

Initial Genes	Optional Genes
ABCA3, ABCB6, ABHD12, ADAMTS18, ADAMTSL4, AGK, ALDH18A1, BCOR, BEST1, BFSP1, BFSP2, CHMP4B, CLN3, COL11A1, COL18A1, COL2A1, COL4A1, COL4A2, CRYAA, CRYAB, CRYBA1, CRYBA4, CRYBB1, CRYBB2, CRYBB3, CRYGB, CRYGC, CRYGD, CRYGS, CTDP1, CYP27A1, CYP51A1, EPG5, EPHA2, ERCC2, ERCC5, ERCC6, ERCC8, EYA1, FAM126A, FOXC1, FOXE3, FTL, FYCO1, FZD4, GALK1, GALT, GCNT2, GFER, GJA1, GJA3, GJA8, HMX1, HSF4, JAM3, LEMD2, LIM2, LONP1, LSS, MAF, MIP, MIR184, MYH9, NDP, NF2, NHS, OCRL, OPA3, P3H2, PAX6, PEX10, PEX11B, PEX16, PEX2, PEX7, PITX2, PITX3, PXDN, RAB18, RAB3GAP1, RAB3GAP2, RDH11, RECQL4, RGS6, RNLS, RRAGA, SC5D, SIL1, SIPA1L3, SIX6, SLC16A12, SLC33A1, TBC1D20, TDRD7, TFAP2A, TMEM70, UNC45B, VIM, VSX2, WDR87, WFS1, WRN, XYLT2	CHMP4B, CRYGB, LIM2, VIM

Table S1C. GeneDx Cataract Panel
CRYBB3, ABCA3, ABHD5, ADAMTSL4, AGK, AKR1E2, ALDH18A1, BCOR, BEST1, BFSP1, BFSP2, CHMP4B, COL11A1, COL2A1, COL4A1, COL4A2, CRYAA, CRYAB, CRYBA1, CRYBA2, CRYBA4, CRYBB1, CRYBB2, CRYGB, CRYGC, CRYGD, CRYGS, CTDP1, CYP27A1, CYP51A1, EBP, EPG5, EPHA2, ERCC2, ERCC5, ERCC6, ERCC8, EYA1, FAM126A, FOXC1, FOXE3, FTL, FYCO1, FZD4, GALK1, GALT, GCNT2, GFER, GJA1, GJA3, GJA8, HMX1, HSF4, JAM3, LIM2, LONP1, LSS, MAF, MAN2B1, MIP, MIR184, MYH9, NDP, NF2, NHS, OCRL, OPA3, PAX6, PEX11B, PEX7, PITX2, PITX3, PXDN, RAB18, RAB3GAP1, RAB3GAP2, RECQL4, RGS6, RNLS, RRAGA, SC5D, SIL1, SIPA1L3, SIX6, SLC16A12, SLC33A1, TBC1D20, TDRD7, TFAP2A, TMEM70, UNC45B, VIM, VSX2, WDR87, WFS1, WRN

Table S1D. Fulgent Cataract panel
ABHD12, AGK, BEST1, BFSP1, BFSP2, CAV1, CHMP4B, CLPB, COL18A1, CRYAA, CRYAB, CRYBA1, CRYBA2, CRYBA4, CRYBB1, CRYBB2, CRYBB3, CRYGB, CRYGC, CRYGD, CRYGS, CTDP1, EPHA2, EYA1, FAM126A, FOXE3, FTL, FYCO1, GALK1, GCNT2, GFER, GJA3, GJA8, HSF4, JAM3, LEMD2, LIM2, LSS, MIP, MIR184, MSMO1, NHS, P3H2, PAX6, PITX3, PXDN, RDH11, SIL1, SIPA1L3, SIX6, SLC16A12, SLC33A1, TDRD7, UNC45B, VIM, WFS1

Table S1E. CD Genomics Gene Panel (can choose which genes you want)
ABCA3, ADAMTSL4, AGK, AKR1E2, ALDH18A1, BCOR, BEST1, BFSP1, BFSP2, CHMP4B, COL11A1, COL2A1, COL4A1, COL4A2, CRYAA, CRYAB, CRYBA1, CRYBA2, CRYBA4, CRYBB1, CRYBB2, CRYGB, CRYGC, CRYGD, CRYGS, CTDP1, CYP27A1, CYP51A1, EOGT, EPHA2, FAM126A, FOXC1, FOXE3, FTL, FYCO1, FZD4, GALK1, GCNT2, GFER, GJA1, GJA3, GJA8, HMX1, HSF4, JAM3, LIM2, LONP1, LSS, MAF, MIP, MIR184, MYH9, NDP, NF2, NHS, OCRL, OPA3, P3H2, PAX6, PITX2, PITX3, PXDN, RAB18, RAB3GAP1, RAB3GAP2, RECQL4, RGS6, RNLS, RRAGA, SC5D, SIL1 , SIPA1L3, SIX6, SLC16A12, SLC33A1, TBC1D20, TDRD7, TFAP2A, TMEM70, UNC45B, VIM, VSX2, WDR87, WFS1, WRN

Table S1F. Blueprint genetics cataract panel
ABCB6, ADAMTS18, ADAMTSL4, AGK, ALDH18A1, BCOR, BFSP1, BFSP2, CHMP4B, COL11A1, COL18A1, COL2A1, COL4A1, CRYAA, CRYAB, CRYBA1, CRYBA4, CRYBB1, CRYBB2, CRYBB3, CRYGC, CRYGD, CRYGS, CTDP1, CYP27A1, DNMBP, EPHA2, ERCC2, ERCC5, ERCC6, ERCC8, EYA1, FAM126A, FOXE3, FTL, FYCO1, FZD4, GALE, GALK1, GALT, GCNT2, GJA1, GJA3, GJA8, HSF4, LEMD2, LIM2, MAF, MIP, MT-ATP6, MT-ATP8, MT-CO1, MT-CO2, MT-CO3, MT-CYB, MT-ND1, MT-ND2, MT-ND3, MT-ND4, MT-ND4L, MT-ND5, MT-ND6, MT-RNR1, MT-RNR2, MT-TA, MT-TC, MT-TD, MT-TE, MT-TF, MT-TG, MT-TH, MT-TI, MT-TK, MT-TL1, MT-TL2, MT-TM, MT-TN, MT-TP, MT-TQ, MT-TR, MT-TS1, MT-TS2, MT-TT, MT-TV, MT-TW, MT-TY, MYH9, NDP, NF2, NHS, OCRL, OPA3, P3H2, PAX6, PITX3, PXDN, RAB18#, RAB3GAP1, RAB3GAP2#, RECQL4, SIL1, SIPA1L3, SLC16A12, SLC33A1, TBC1D20, TDRD7, TFAP2A, TMEM70, VIM, VSX2, WFS1, WRN, XYLT2

Supplementary Table S2. Individual Patient Demographics

Patient #	Race / Ethnicity	Systemic Illness/Syndrome	Ocular Findings	Type of Cataract	Family History	Age at Surgery
1	White/NH	WAGR syndrome, pineal gland hypoplasia, tachycardia with prolonged QTc, persistent left superior vena cava, Wilms tumor in one kidney	Congenital ptosis OD, aniridia, glaucoma, intermittent exotropia, nystagmus, amblyopia OD, absent cranial nerve VI bilaterally	OU: Anterior polar cataracts	No	Unknown
2	Other/Hispanic	Developmental delay	Optic nerve temporal pallor with sloping OU, mild attenuation of vessels OU, mild intermittent exotropia	OU: Scattered flecks with 1+ posterior subcapsular cataract and trace cortical changes OU	No	>10 years
3	White/NH	2 vessel cord, hypotonia at birth, small for gestational age, small kidneys, left mild pelviectasis, sacral dimple, ventral hypospadias, left pulmonary artery hypoplasia, epilepsy, hypospadias, micrognathia, globally delayed	No	OU: Large nuclear cataracts	No	<1 year
4	White/NH	Micrognathia, dysmorphic appearance due to in-utero constriction	Physiologic anisocoria	OU: Posterior lenticular opacity <1 mm	No	Unknown
5	Other/Hispanic	Cleft lip/palate, poor feeding, small VSD and PFO, prolonged QTc interval, hearing loss	Left congenital ptosis	OU: Cortical cataracts	No	Unknown
6	White/NH	Microcephaly, bilateral sensorineural hearing loss, reflux, developmental delay, poor growth	OD microphthalmia, OU chorioretinal colobomas, OD optic nerve coloboma, OU iris colobomas	OD: Small nuclear cataract, OS: Clear	No	Unknown
7	Other/Hispanic	Thyroglossal cyst, learning disorder, ADHD, expressive language disorder	Intermittent esotropia	OD: Nasal posterior polar cataract, not in visual axis, OS: Posterior polar,	No	1-10 years

				nuclear and cortical cataract		
**8**	White/NH	Developmental delays/ASD	Aphakic glaucoma OU, esotropia	OU: cataracts of unknown description	No	<1 year
**9**	White/NH	Microcephaly, developmental delay, CNS calcifications	OU mild microphthalmia, persistent pupillary membrane, esotropia	OU: Dense posterior subcapsular polar cataracts in center of visual axis, OS: more dense cataract	No	<1 year
**10**	Other/NH	Heterotaxy (midline liver, dextrocardia with VSD, horseshoe kidney, small size and developmental delay), imperforate anus	Intermittent esotropia	OU: White cataract	No	< 1 year
**11**	Other/Hispanic	Congenital heart disease (VSD, coarctation of aorta), respiratory distress, IUGR, horseshoe kidney, extremity edema, tethered cord, labial adhesion, hemangioma, developmental delays, hypotonia, hypothyroidism, hypocalcemia, hyperinsulinism, non-occlusive thrombus, delayed growth	Euryblepharon, orbital lipodermoid OS	OU: Lamellar cataracts	No	1-10 years
**12**	White/NH	Recurrent respiratory infections, inguinal hernia, delayed early milestones, then normal intelligence	Tilted optic nerves	OU: Ectopia lentis (superior dislocation) cortical haze, proliferation of anterior lens capsule	No	>10 years
**13**	White/NH	Hearing loss, developmental delays	Small esotropia	OU: Posterior subcapsular cataracts	No	>10 years
**14**	Other/Hispanic	Small ASD, bilateral SNHL (hearing aids in place, knows sign language), microcephaly, dysmorphic facies,	Exotropia, possible bilateral myelinated NFL	OD: 1-2+ Cortical with lamellar components, 1+	No	>10 years

		cleft palate, hypotonia, moderate cognitive deficits, pituitary dwarfism		PSC centrally, OS: 2+ Central PSC, 2-3+ cortical with lamellar components		
**15**	Black/NH	Epilepsy, developmental delays, left sensorineural hearing loss, failure to thrive, short stature, growth hormone deficiency, asthma, pes planus, eczema, hydrocephalus s/p VP shunt, born at 25 weeks, ADHD, ASD, microcephaly - felt by genetics issues most likely from prematurity	Bilateral optic atrophy/cupping, mild nystagmus, microstrabismus, history of ROP without treatment	OD: Clear, OS: Small left anterior polar cataract	No	Unknown
**16**	White/NH	Hypotonia, dysmorphic features, lowset, protruding ears, high arched palate, mild motor delays	OD: Microphthalmia, aphakic glaucoma, OS: otherwise normal, variable strabismus	OD: Central 5-6 mm dense nuclear and posterior opacification, OS: very minimal cortical haze/posterior opacification with wispy appearance	No	<1 year
**17**	Other/NH	Muscle eye brain disease, static encephalopathy, severe global developmental delay, ventriculomegaly, right side of face slightly smaller, right kidney smaller than left, persistent left superior vena cava draining into coronary sinus	OD: Microphthalmia, PHPV, posterior synechiae, OS: optic nerve hypoplasia, blunted fovea, abnormal blood vessels	OD: White cataract, possible PHPV, OS: possibly traumatic cataract	No	Unknown
**18**	Other/Hispanic	Hydrocephalus, epilepsy, CP, developmental delay, digital anomalies (missing distal part of fingers and toes 2-5 bilaterally), cortical malformations, possible absence of septum pellucidum, neurogenic bladder	Heterochromia (OD mid and peripheral iris atrophy/thin stroma, OS brown), OD: microphthalmia, gliosis off nerve, large chorioretinal scar vs coloboma temporal	OD: Inferior cortical cataract encroaching on visual axis	No	Unknown

			to nerve, nerve is dragged, esotropia/exotropia			
19	Black / Hispanic	None	None	OU: <1 mm bilateral anterior polar cataracts, otherwise clear	No	Unknown
20	White / Hispanic	None	Esotropia, latent nystagmus	OU: Diffuse nuclear cataract	No	<1 year
21	Asian / NH	Poor growth	Microphthalmia, esotropia	OU: Nuclear cataracts (OD more dense than OS)	Yes (2 sisters with congenital cataracts, no other known affected relatives)	<1 year
22	Other / Hispanic	Borderline small head	Anterior segment dysgenesis (posterior embryotoxon, iris atrophy, PPM, persistent tunica vasculosa lentis, microphthalmia), pendular nystagmus	OD: Anterior polar with nuclear cataract and posterior vacuole (possible Lenticonus)), OS: anterior polar with anterior subcapsular/cortical, scattered nuclear/cortical cataract, prominent anterior lenticonus	No	<1 year
23	White / NH	Nance-Horan Syndrome, Penal-scrotal webbing, Buried penis	Aphakic glaucoma OD, intermittent exotropia, latent nystagmus	OU: Cortical cataracts	Yes (mother with mild cataracts, maternal grandmother possible cataracts, no other	<1 year

					known affected relatives)	
**24**	White / NH	None	OU: Partial aniridia	OU: Small anterior polar cataracts	No	Unknown
**25**	Asian / NH	Brachycephaly, hypogonadotrophic hypogonadism, webbing, pharyngeal dysphagia, hypotonia, global developmental delays	OU: Small optic nerves, likely cortical visual impairment	OU: White cortical cataracts	No	<1 year
**26**	Unknown/ NH	None	None	Unknown	No	>10 years
**27**	White/NH	Failure to thrive, reflux	OU: Aniridia, nystagmus	OD: 3+ Lamellar cataract, OS: Clear	No	Unknown
**28**	White / NH	None	Anterior segment dysgenesis (partial aniridia, PPM, tunica vasculosa lentis) OS only, foveal hypoplasia OS, congenital optic disc anomaly OS, exotropia OS, chorioretinal coloboma OS, microphthalmia OS	OD: Clear, OS: Megalolenticonus , central posterior cortical/capsular cataract with fine PFV stalk to ON	No	<1 year
**29**	Black / NH	None	Nystagmus, bilateral colobomas of iris, nerves, chorioretinas, high myopia	OD: Small inferior wedge shaped opacity, OS: Clear	No	Unknown
**30**	White/NH	None	None	OU: Mixed cataracts with calcium	No	<1 year
**31**	Other / NH	None	None	OU: Lamellar/nuclear cataracts	No	1-10 years
**32**	Other / Hispanic	Microcephaly, developmental delays, cleft palate, dysmorphic features, hypotonia, micrognathia, triphalangeal thumbs, hydrocephalus, posterior fossa cyst, possible seizures, sensorineural hearing loss, dysphagia, hip	haze of cornea superiorly OU, microphthalmia (cornea <10 mm) OU, crowded optic discs but no swelling, OS vertically elongated ON, anomalous vascular pattern from	OU: <1 mm Anterior polar cataracts, otherwise clear	No	Unknown

		dysplasia, PFO/PDA, pigmentary abnormalities of skin	nerves, otherwise normal funuds, moderate myopia/astigmatism			
**33**	White / NH	Peripheral neuropathy, lung disease, intellectual disability, failure to thrive, hypotonia, decreased bone density	Exotropia, possible optic nerve pallor	OU: Nuclear cataract	No	1-10 years
**34**	White / NH	Pachygyria, benign childhood seizure disorder, autism spectrum disorder, poor weight gain, posteriorly thin corpus callosum, eczema	Intermittent esotropia	OU: Diffuse cortical cataracts	No	1-10 years
**35**	Other / Hispanic	Ventriculomegaly, Dandy Walker variant, global developmental delay, epilepsy	Optic nerve pallor, attenuated retinal vessels, cortical visual impairment, exotropia	OU: Posterior subcapsular cataracts	No	Unknown
**36**	White / NH	VSD, gait abnormalities, epilepsy	OU: Aphakic glaucoma, esotropia, nystagmus	OU: Cataracts (type unknown)	No	<1 year
**37**	White / NH	OFCD syndrome - dysmorphic features, dysphagia, club foot, PDA and ASD s/p cardiac surgery, vertical ridging of incisors, laryngeal cleft, delayed dentition, neurogenic bladder, dystrophic toenails, fatty filum/tethered cord, developmental delays	OU: Progressive high myopia, congenital ptosis upper eyelids, exotropia, DVD	OU: Posterior cortical cataracts and posterior lenticonus	No	<1 year
**38**	White / NH	Congenital muscular dystrophy, microcephaly, periventricular white matter changes, mild cognitive deficits	OD: Clinical optic atrophy, OU: Small optic nerves on MRI	OD: Millet seed opacities throughout, mostly posterior without sclerosis of nucleus, OS: white cataract	No	>10 years
**39**	Other / Hispanic	Hydrocephalus s/p VP shunt, Vein of Galen malformation, Meckel's diverticulum, mildly dilated aortic root and main pulmonary artery, tiny muscular VSD, pectus excavatum, joint hypermobility, port wine stains, hyperinsulinemia, hypertriglyceridemia	OU: Hgh myopia, questionable history of exudative retinopathy	OD: Tiny round posterior cortical opacity, OS: Small central cortical spoke opacity	No	>10 years

**40**	Asian / NH	Sensorimotor neurpathy, short stature, secondary amenorrhae, spinocerebellar ataxia	OU: High myopia	OD: 3mm posterior polar cataract, no stalk, OS: 3 mm posterior polar cataract, no stalk, few small cortical spokes	No	>10 years
**41**	White / Hispanic	Pigmentary mosaicism, toe deformity, laryngomalacia with mild subglottic narrowing, many pigmented nevi, failure to thrive, speech delay	None	OD: Anterior polar cataract, OS: Clear	Yes (mother with cataracts, no other known affected relatives)	>10 years
**42**	White / Hispanic	ASD with PDA, torticollis, hypotonia with hypertronia, developmental delay	OD: Mild microphthalmia, myopic fundus, grayish nerve, staphyloma, myopic/blonde fundus, severe myopia, esotropia; OS: Normal	OD: Posterior subcapsular cataract, not in visual axis, OS: Clear	No	Unknown
**43**	Other/Hispanic	DiGeorge Syndrome, recurrent OM, multiple dental anomalies, AV canal, subaortic stenosis s/p surgery, hx BPD, asthma, hx dysphagia, EOE, polydactyly of left hand, abnormal digits on both feet with marked varus deformmity of toes, mild learning difficulties, hypogammaglobulinemia, history of poor weight gain	OU iris nevi, IXT	OD: Cortical lamellar superotemporal cataract not visually significant, OS: Clear	No	>10 years
**44**	Other/Hispanic	Feeding intolerance, hypertonia, some developmental delays	Nystagmus, esotropia	OU: Mixed lamellar/nuclear cataracts obstructing posterior view of fundus	No	<1 year
**45**	Other/Hispanic	Micrcocephaly, epilepsy, global developmental delays, persistent feeding issues, hypotonia	OU: Possible temporal pallor of optic nerves	OU: Diffuse opacification of	No	1-10 years

				anterior capsule and cortex		
46	Other/Hispanic	Walker-Warburg syndrome: epileptic encephalopathy, muscle weakness, hydrocephalus, chronic respiratory failure	OU: microphthalmia, persistent tunica vasculosa lentis with no view posteriorly B scan with thick stalks from lens to nerve - likely bilateral retinal detachment/PFV, corneal anomaly, likely Peters OD (cornea/lens touch OD	OU: Posterior cataract, dense/retrolental membrane, suggestive of PFV	No	Unknown
47	Black/NH	Complicated birth history (lack of prenatal care, intrauterine drug exposure, IVH s/p surgery, neonatal seizures, neonatal cerebral cystic leukomalacia, jaundice), spastic hemiplegic cerebral palsy, sensorineural hearing loss, cholestasis and transaminitis	OU: Hypopigmented foveal scar, esotropia	OU: Diffuse nuclear cataracts	No	<1 year
48	Black/NH	Epilepsy, neurodevelopmental delay, fifth finger flexion deformity, congenital ankle valgus, mild dysmorphic features	Esotropia	OD: Non-visually significant cortical cataract peripherally, OS: Intumescent cataract	No	1-10 years
49	Other/Hispanic	Lennox Gastaut Syndrome, intractable epilepsy, dysphagia and global developmental delay, nocturnal hypoxemia, pectus excavatum	Nystagmus, cortical visual impairment	OU: Lamellar cataract with cortical component (likely visually significant but same behavior so no surgery done yet)	No	Unknown
50	White/NH	Failure to thrive, delayed postnatal growth, neonatal hypoglycemia, developmental delay, hypotonia, hypothyroid, upper airway resistance syndrome, NASH, Legg-Calvé-Perthes disease	Esotropia	OU: Dense white cataracts	No	1-10 years

**51**	White/NH	Developmental delay, stroke, scoliosis, osteopenia, microcephaly	Bilateral lagophthalmos, exotropia	OD: White cataract, OS: Mild PFV, not visually significant	No	>10 years
**52**	White/NH	Paraganglioma of carotid body, congenital chordee, hsereditary paraganglioma syndrome	None	OD: 0.1 mm Focal opacity over posterior capsule, nasal to visual axis, not visually significant, OS: Clear	Yes (father with early cataracts, no other known affected relatives)	>10 years

"NH" = Not Hispanic. OD = right eye, OS = left eye, OU = both eyes. "Yes" for Family History includes patients with family history of congenital cataracts. Age at surgery is divided into 4 groups: (1) patients with severe cataracts in infancy requiring surgery <1 year old, (2) patients with cataracts that progress to becoming visually significant after infancy but still during the amblyogenic period (1-10 years old), (3) patients with cataracts that either remain visually insignificant or require surgery after the amblyogenic period (>10 years old), and (4) patients that are <10 years old currently and have not needed surgery yet ("Unknown").

"WAGR" = Wilms tumor, aniridia, genitourinary anomalies, and intellectual disability, "VSD" = ventricular septal defect, "PFO" = patent foramen ovale, "ADHD" = attention-deficit/hyperactivity disorder, "ASD" = autism spectrum disorder, "CNS" = central nervous system, "IUGR" = intrauterine growth restriction, "SNHL" = sensorineural hearing loss, "VP" = ventriculoperitoneal, "CP" = cerebral palsy, "PDA" = patent ductus arteriosus, "OFCD = oculofaciocardiodental syndrome, "IVH" = intraventricular hemorrhage, "NASH" nonalcoholic steatohepatitis

"ROP" = retinopathy of prematurity, "PHPV" = persistent hyperplastic primary vitreous, "PPM" = persistent pupillary membrane, "DVD" = dissociated vertical deviation

Supplementary Table S3. Pathogenic (P), Likely Pathogenic (LP), and Variants of Uncertain Significance (VUS) Identified in Initial Genetic Testing of Patients with Congenital Cataracts

Patient #	Gene	Transcript	C.	P.	Zygosity	Highest Allele Frequency	Classification	Reclassification
19	*CAPN15*	NM_005632.2	c.2432C>T	p.Ala811Val	Heterozygous	0.017%, Eastern Asian	VUS	VUS
	CYP27A1	NM_000784.3	c.1343G>A	p.Arg448His	Heterozygous	0.038%, African	VUS	VUS
	FYCO1	NM_024513.3	c.3323A>G	p.Lys1108Arg	Heterozygous	0.47%, African	VUS	VUS
20	*CRYBB2*	NM_000496.2	c.158T>C	p.Leu53Pro	Heterozygous	Novel variant	VUS	VUS
21	*INPP5B*	NM_005540.2	c.730C>T	p.Arg244*	Heterozygous	Novel variant	VUS	VUS
	LTBP2	NM_000428.2	c.1258G>A	p.Ala420Thr	Heterozygous	0.0097%, South Asian	VUS	VUS
	POMT2	NM_013382.5	c.1754C>G	p.Thr585Arg	Heterozygous	Novel variant	VUS	VUS
22	*PAX6*	NM_000280.4	c.151G>A	p.Gly51Arg	Heterozygous	Unknown (not novel)	LP	P
23	*NHS*	NM_198270.3	c.322G>T	p.Glu108*	Hemizygous	Unknown (not novel)	P	P
25	*RAB3GAP1*	NM_012233.2	c.452_453del	p.Ser151Cysfs*38	Homozygous	Novel variant	LP	LP
	RAB3GAP1	NM_012233.2	c.1198T>C	p.Ser400Pro	Homozygous	0.006%, Latino	VUS	VUS
	BFSP1	NM_001195.4	c.1574A>G	p.Gln525Arg	Heterozygous	0.0027%, European (Non-Finnish)	VUS	VUS

	BFSP1	NM_001195.4	c.1480G>A	p.Gly494Arg	Heterozygous	0.34%, South Asian	VUS	VUS
	ADAMTS10	NM_030957.3	c.557G>A	p.Arg186His	Heterozygous	0.13%, South Asian	VUS	VUS
	AGPS	NM_003659.3	c.220A>T	p.Thr74Ser	Heterozygous	0.04%, South Asian	VUS	LB
	ERCC1	NM_202001.2	c.818T>C	p.Leu273Ser	Heterozygous	Novel variant	VUS	VUS
**26**	*COL18A1*	NM_030582.3	c.2300C>T	p.Ala767Val	Heterozygous	0.16%, European (Non-Finnish)	VUS	VUS
	GALK1	NM_000154.1	c.814A>G	p.Lys272Glu	Heterozygous	0.0030%, European (Non-Finnish)	VUS	VUS
**27**	*PAX6*	NM_001368890.2	c.358del	p.Val120Cysfs*4	Heterozygous	Unknown	P	LP
**30**	*CRYAA*	NM_000394.3	c.347G>A	p.Arg116His	Heterozygous	Unknown (not novel)	P	P
	CRYBB1	NM_001887.3	c.300C>T	Silent	Heterozygous	0.03%	VUS	VUS
**31**	*ERCC2*	NM_000400.3	c.1906C>T	p.Arg636Trp	Heterozygous	0.0098%, South Asian	VUS	VUS
	ERCC2	NM_000400.3	c.58G>A	p.Glu20Lys	Heterozygous	Novel variant	VUS	VUS
**32**	*PHACTR4*	NM_001048183.1	c.1868T>C	p.Leu623Pro	Mosaic	Novel variant	VUS	VUS
**33**	*ALDH18A1*	NM_002860.3	c.482G>A	p.Cys161Tyr	Heterozygous	Novel variant	LP	LP

**34**	*DYNC1H1*	NM_001376.4	c.4868G>A	p.Arg1623Gln	Heterozygous	Novel variant	LP	P
	LRP5	NM_002335.2	c.2384A>G	p.Asn795Ser	Heterozygous	0.002-0.01%, European	VUS	VUS
**35**	*KIF1A*	NM_001244008.2	unavailable	unavailable	Heterozygous	Unknown	P	n/a
**36**	*BCOR*	NM_017745.5	c.1857dupC	p.Ser620GlnmfsX25	Heterozygous	Novel variant	LP/P	LP
	ATP2A3	NM_174953.2	c.2021G>A	p.Arg674His	Heterozygous	0.49%, European	VUS	LB
**37**	*BCOR*	NM_017745.5	c.3487C>T	p.Arg1163Stop	Heterozygous	Novel variant	LP/P	P
**40**	*SYNE1*	NM_033071.3	c.19010T>A	p.Leu6337Ter	Homozygous	Novel variant	P	LP
	AAAS	NM_015665.5	c.43C>A	p.Gln15Lys	Heterozygous	Unknown (not novel)	P	P
**41**	*PIK3R1*	NM_181523.2	c.18C>G	p.Tyr6Ter	Heterozygous	Novel variant	VUS	LP
**42**	*RARB*	NM_000965.4	c.844G>A	p.Gly282Ser	Heterozygous	Novel variant	LP	LP
**43**	*WDPCP*	NM_015910.5	c.1594_1596del	p.Leu532del	Heterozygous	0.0016%	VUS	VUS
	WDPCP	NM_015910.5	c.2125G>A	p.Gly709Arg	Heterozygous	0.0004%	VUS	VUS
**46**	*POMT1*	NM_007171.3	c.1241+1G>A	NA	Homozygous	0.0033%, South Asian	P	P
**47**	*NOTCH2*	NM_024408.4	c.4999G>T	p.Val1667Phe	Unknown	Unknown (not novel)	VUS	LB
**48**	*CSNK2B*	NM_001320.6	c.500T>C	p.Leu167Pro	Heterozygous	Novel variant	LP	LP
	KCNB1	NM_004975.2	c.1169G>T	p.Gly390Val	Heterozygous	Novel variant	LP	LP
**49**	*NR4A2*	NM_006186.4	c.325dup	p.Gln109Profs*3	Heterozygous	0.0004%	P	P

	DYNC1H1	NM_001376.4	c.11806G>A	p.Val3936Met	Heterozygous	0.01%, Latino	VUS	LB
	PNKP	NM_007254.3	c.416G>A	p.Arg139His	Heterozygous	0.3%, European	VUS	LB
	SCN2A	NM_021007.2	c.2149+3A>C	unknown	Heterozygous	Novel variant	VUS	VUS
**50**	*GRB10*	NM_005311.4	c.1456G>A	p.Val486Met	Heterozygous	Unknown	LP/P	VUS
**51**	*NGLY1*	NM_018297.3	c.1201A>T	p.Arg401Ter	Homozygous	Unknown	LP/P	P
**52**	*SDHD*	NM_0003002.4	c.53dupC	p.Leu19SerfsX50	Heterozygous	Unknown (in father, not otherwise reported)	LP/P	P
